# Multimer Detection System-Oligomerized Amyloid Beta (MDS-OA*β*): A Plasma-Based Biomarker Differentiates Alzheimer's Disease from Other Etiologies of Dementia

**DOI:** 10.1155/2022/9960832

**Published:** 2022-05-02

**Authors:** Jacqueline Cotoong Dominguez, Jeryl Ritzi Tan Yu, Ma Fe De Guzman, Encarnita Ampil, Anne Cristine Guevarra, Ma. Lourdes Joson, Macario Reandelar, Ma. Socorro Martinez, Antonio Ligsay, Ferron Ocampo, SangYun Kim

**Affiliations:** ^1^St Luke's Medical Center, Institute for Neurosciences, Philippines; ^2^Institute for Dementia Care Asia, Philippines; ^3^St. Luke's College of Medicine William H. Quasha Memorial, Philippines; ^4^Research and Biotechnology Division, St. Luke's Medical Center, Philippines; ^5^Department of Neuroscience and Behavioral Medicine, Faculty of Medicine and Surgery, University of Santo Tomas, Philippines; ^6^Far Eastern University, Dr. Nicanor Reyes Medical Foundation, Philippines; ^7^Our Lady of Fatima University, Philippines; ^8^New Era University College of Medicine, Philippines; ^9^College of Science and the Graduate School, University of Santo Tomas, Philippines; ^10^Department of Neurology, Seoul National University College of Medicine & Neurocognitive Behavior Center, Seoul National University Bundang Hospital, Seongnam, Republic of Korea

## Abstract

With emerging amyloid therapies, documentation of the patient's amyloid status to confirm the etiology of a clinical diagnosis is warranted prior to instituting amyloid-based therapy. The Multimer Detection System-Oligomeric Amyloid-*β* (MDS-OA*β*) is a noninvasive blood-based biomarker utilized to measure A*β* oligomerization tendency. We determined the difference in MDS-OA*β* ratio across the groups: (a) no cognitive impairment or subjective cognitive impairment (NCI/SCI), (b) Alzheimer's disease (AD), (c) non-AD, and (d) mixed Alzheimer's disease-Vascular dementia (AD-VaD). MDS-OA*β* level was not significantly different between AD and mixed AD-VaD, but both groups were significantly different from the NCI/SCI and from the non-AD group. An MDS-OA*β* level of >1 could potentially indicate clinical variants of AD or mixed pathology (AD-VaD).

## 1. Introduction

Alzheimer's disease (AD) is a progressive neurodegenerative disorder that commonly affects the elderly [1], accounting for up to 80% of dementias, [2] with a heavy socio-economic burden [3, 4]. The diagnosis of AD at preclinical stages is critical, because treatment after the onset of clinical symptoms cannot halt or reverse disease progression. Revisions in the diagnostic criteria of dementia due to AD based on the National Institute on Aging and the Alzheimer's Association (NIA-AA) included documentation of brain amyloid via biomarkers [5]. Among these, the most well established and validated are cerebrospinal fluid amyloid-*β* 42 (A*β*_42_) (CSF A*β*_42_) and PET amyloid imaging [5, 6]. However, these biomarkers are rarely utilized in the clinical setting as they are invasive and expensive and have limited accessibility to patients [7], leading to the development of blood-based biomarkers [8–10] which can provide minimally invasive and inexpensive methods for early screening and diagnosis of AD.

Brain amyloidosis is a critical hallmark of AD, and its pathologic development starts 10-20 years prior to its clinical manifestation and onset of cognitive symptoms [5, 11]. There is accumulating evidence that oligomerized A*β* (OA*β*) is the most neurotoxic among different A*β* types and that aggregation of these oligomers is toxic in vivo [12]. Compared to amyloid-*β* plaque loads, oligomerized amyloid-*β* (OA*β*) was found to have higher correlation with presence and severity of cognitive symptoms [13, 14]. Hence, A*β* oligomers played a key role in pathogenesis and prediction of AD diagnosis [15]. Recently, an enzyme-linked immunosorbent assay, the Multimer Detection System-Oligomeric Amyloid-*β* (MDS-OA*β*), was utilized to detect A*β* oligomerization in plasma, correlating well with other clinical biomarkers such as CSF A*β*_42_ and amyloid PET [16]. It measures the dynamic change of plasma oligomeric A*β* concentration, which is higher in AD patients compared to older adults with normal cognition [15]. MDS-OAB had shown a higher diagnostic accuracy in identifying AD from controls, compared with other amyloid biomarkers that evaluates plasma OAB concentrations [17–19]. However, limited studies have compared OA*β* levels among individuals with AD and other dementia types [15, 17]. Given that underlying etiologies of cognitive decline impact emerging therapies that are target-specific, clinicians need clinically valid and cost-effective biomarkers that can be utilized to provide accurate diagnosis of dementia etiology. The aim of this study is to investigate whether MDS-OA*β* could assist in determination of AD from dementia of other etiologies.

## 2. Materials and Methods

### 2.1. Subjects

Participants were enrolled at the Memory Center of St. Luke's Medical Center Global City from January 2018 to March 2019. All participants underwent clinical evaluation and detailed neuropsychological assessment, cranial magnetic resonance imaging (MRI), and MDS-OA*β* blood test (first made available in the Philippines in November 2017). The inclusion criteria were as follows: (1) adult patients with subjective report of cognitive decline or memory impairment; (2) diagnosed and categorized by a dementia specialist into one of the following: (a) no cognitive impairment/subjective cognitive impairment (NCI/SCI): presence of persistent cognitive symptoms without evidence of impairment in psychometric tests; (b) dementia, based on the DSM-IV TR criteria [20] and further classified into the following clinical diagnosis: AD, NINCDS-ADRDA criteria [5]; mixed AD-VaD, coexistence of AD and vascular dementia (VaD) as defined in NINDS-AIREN criteria [21]; and non-AD dementia, including frontotemporal dementia, Lewy body disease, and Parkinson dementia; (3) underwent comprehensive neuropsychological assessment (clinical dementia protocol) at the Memory Service clinic; and (4) MDS-OA*β* result available. Diagnosis was made at the end of the clinical assessment without knowing the result of the MDS-OA*β* blood test.

Each eligible participant was assigned a study code, and data on demographic characteristics, Clinical Dementia Rating (CDR) score, and etiologic diagnosis based on the clinician's assessment were recorded. MDS-OA*β* blood test for amyloid oligomerization tendency was done as per physician's request, and the MDS-OA*β* levels were obtained. The data collection forms were documented by a nurse clinician and reviewed by a neurologist. Data were encoded independently, and inconsistencies were double-checked with the data source to ensure data accuracy.

Sample size was calculated using the test of hypothesis for the difference between mean and standard deviation (SD) of MDS-OA*β* among patients with MCI versus those healthy normal control. Assuming that mean and SD of MDS-OAß among MCI patients is 0.964 ± 0.098 and among healthy normal controls, 0.904 ± 0.130 [22], with an alpha error of 5%, power of 95%, and a one-tailed alternative hypothesis, sample size required is 23 per group, for a total of 92 for 4 groups.

### 2.2. MDS-OA*β* Assay Description and Procedure

The inBlood™ oligomerized A*β* (OA*β*) test (PeopleBio Inc., Republic of Korea) was used to quantify MDS-OA*β* values in the plasma from the subjects. This test is based on the Multimer Detection System (MDS), which is a modified enzyme-linked immunosorbent assay (ELISA) using epitope-overlapping detection antibodies specific for the N-terminus of A*β* for the selective detection of OA*β* over A*β* monomers.

Prior to the assay, aliquots of plasma samples were thawed at 37°C for 15 min. As indicated in the assay protocol of the inBlood™ OA*β* test, PBR-1 (synthetic A*β* made by PeopleBio Inc.) was spiked into plasma, and the mixture was incubated at 37°C for 48 hours. The incubated plasma sample mixture and serially diluted standard samples were added to each well of the plates. The plates were incubated at about 20-25°C for 1 hour. After washing three times with washing buffer, W02-HRP antibody (Absolute Antibody Ltd., UK) was added to the wells, and the plates were incubated for 1 hour at about 20 to 25°C. To increase the sensitivity of detection, 100 *μ*l/well of enhanced chemiluminescence substrate solution (Rockland Immunochemicals Inc., USA) was added, and the Relative Luminescence Unit (RLU) signal was detected using a multispectrophotometer. Dilutions providing signal in the linear range of the standard curves were used for the conversion to RLU values to determine the concentration of oligomerized A*β*.

### 2.3. Statistical Analysis

Data was analyzed using Statistical Program for Social Sciences version 23 (SPSS Inc.: IBM). Differences in patients' clinical characteristics across different etiological diagnosis were analyzed using Pearson Chi-square test for categorical variables (i.e., sex, age groups, and CDR severity stages), and one-way analysis of variance (ANOVA) was used for continuous variables. Two-way ANOVA was used to determine the difference in MDS-OA*β* ratio of patients grouped by age and etiological diagnosis. LSD and Bonferroni tests were used for post hoc analysis. Significance level was set at *p* ≤ 0.05 (95% confidence interval).

The conduct of this study was guided by the principles of Good Clinical Practice and in accordance with local regulations and was approved by the St. Luke's Institutional Ethics Review Committee (SL-20192).

## 3. Results

**Figure 1:**
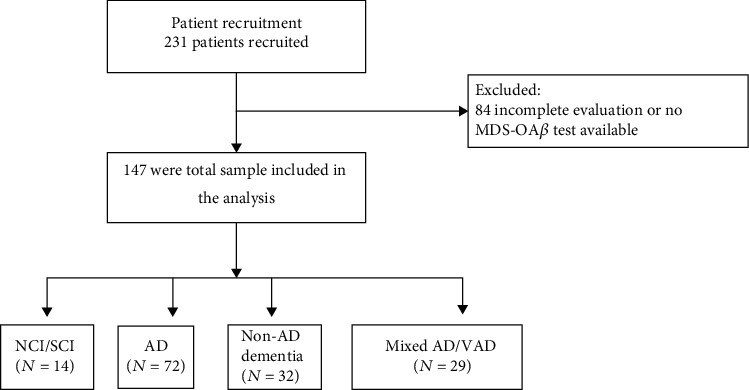
Study flow-chart.

A total of 231 patients were recruited. Of these participants, 84 were excluded due to incomplete evaluation, resulting in 147 patients included in the study (Figure 1). Among those included, majority were females (62.6%) with a mean age of 75.0 ± 10.0 years (age ranged from 38 to 94 years old; Table 1). The most prevalent clinical diagnosis was AD (49.0%), followed by non-AD dementia (21.8%), mixed AD-VaD (19.7%), and NCI/SCI (9.5%). Clinical characteristics were compared across clinical diagnosis for cognitive impairment and dementia. Results showed no significant difference in sex distribution among the four clinical diagnosis (*p* = 0.66). On the other hand, age groups and CDR stage distribution were significantly different across different etiologic diagnosis (*p* < 0.001; Table 1). The age group distribution among the NCI/SCI group was younger compared among patients with AD, non-AD, and mixed AD-VaD. In terms of severity, more than half of the patients were at the earliest stage of cognitive impairment or dementia (CDR = 0.5; 57.1%), followed by mild (CDR = 1; 24.5%), moderate (CDR = 2; 8.2%), and severe (CDR = 3; 4.1%) dementia stages in decreasing order (Table 1).

**Figure 2:**
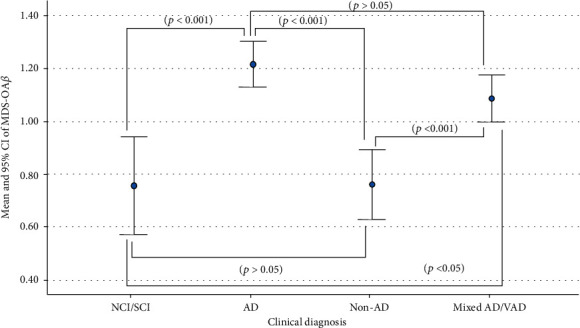
Comparison of the mean and 95% CI of MDS-OA*β* per clinical diagnosis.

The MDS-OA*β* PT/C ratio was significantly different among groups (*p* < 0.001; Table 1). Consistently, the average oligomerized amyloid-*β* (OA*β*) levels were highest among patients with AD diagnosis, followed by mixed AD-VaD and lowest in the NCI/SCI group (Figure 2). Post hoc analysis revealed that OA*β* levels of patients with AD and mixed AD-VaD diagnosis were not significantly different from each other (*p* > 0.05) but significantly different from patients with NCI/SCI (*p* < 0.001) and non-AD (*p* <0.01) diagnosis. There was only marginally significant difference when MDS-OA*β* patient/control (PT/C) ratio was compared across different age groups (≤59, 60-69, 70-79, and ≥80 years old) among four etiologic diagnosis (*p* = 0.07, Table 1), indicating MDS-OA*β* level does not significantly vary based on age groups but more significantly on etiologic cause of cognitive impairment and dementia.

## 4. Discussion

In this study, we found that MDS-OA*β* could differentiate between dementias due AD versus non-AD etiologies. Majority of the patients in this study were in the mild stage with Clinical Dementia Rating (CDR) 0.5 and 1, indicating that MDS-OA*β* could detect AD. These findings have several implications. Given the long preclinical stage of AD, patients would benefit from early diagnosis prior to the onset of symptoms. This is consistent with results from a study by Lee et al. in which MDS-OA*β* was shown to be a useful screening tool for individuals in the MCI stage [23]. MDS-OA*β* also had some correlation with brain volume reduction consistent with AD [24] and showed correlation with decline in memory performance [25], further supporting its utility as a noninvasive biomarker for AD. In a study by Youn et al., MDS-OA*β* was able to validate the clinical diagnosis of AD when compared to normal controls (sensitivity of 100% and specificity of 92.31%) [15].

The level of OA*β* could provide valuable information with regard to the stage or AD progression. MDS-OA*β* measures the oligomerization tendency of A*β*, and it was postulated to correspond to the derivative of the sigmoid function of A*β* accumulation [15]. Studies have shown that oligomer concentrations were higher in MCI or early stage AD [26]. Therefore, an MDS-OA*β* level of >1 in NCI/SCI subjects can correspond to the preclinical stage of AD. As the disease progresses, there is eventual attenuation in the expression of this biomarker. Hence, low OA*β* levels (<0.5) among AD patients are highly suggestive of late-stage AD. A possible explanation could be that the concentrations of biomarkers associated with AD pathogenesis, including OA*β*, show decreasing trend after symptomatic disease progression, denoting slowing of the neurodegenerative process [15]. Moreover, low levels of amyloid in AD patients could also correspond to limbic-predominant age-related TDP-43 encephalopathy (LATE), which is seen in advanced elderly patients with features similar to AD. However, the underlying neuropathology for LATE is characterized by the presence of TDP-43 protein inclusion bodies in the cytoplasm and accumulation of hyperphosphorylated TDP-43 in nuclei and cell processes of neurons [27]. Lastly, patients with non-AD dementia are expected to have low levels of OA*β*; therefore, an MDS-OA*β* level of >1 in these patients could potentially indicate clinical variants of AD or mixed pathology.

In clinical practice, patients with cognitive decline are assessed using tools such as the Mini Mental Status Exam (MMSE) and Montreal Cognitive Assessment (MoCA). When equivocal, additional neuropsychological evaluation is often warranted [1]. The incorporation of CSF A*β*_42_ and PET amyloid biomarkers in the revised diagnostic criteria of Alzheimer's disease (NIA-AA) [5] has resulted in diagnostic and management shifts [28]. However, amyloid imaging biomarkers are not always a feasible option in regions where they are unavailable or unaffordable. In this setting, the underlying etiology of the dementia will best be ascertained via a biomarker that is noninvasive, affordable, and readily available with a high discriminative accuracy.

In addition to clinical diagnosis, this biomarker can also impact on the development of disease modifying therapies (DMT), which aims to delay disease progression and possibly reverse AD [22, 29, 30]. Clinical trials of these DMTs, which include monoclonal antibodies against A*β*, such as aducanumab, solazenumab, and bapineuzumab, often require documentation of amyloid on PET imaging on enrolled subjects [31].

Amyloid PET, which represents A*β* fibrillar morphology, and MDS-OA*β*, which shows oligomerization tendency, reflect different aspects of A*β* pathology, but in lieu of PET, MDS-OA*β* can be utilized to document amyloid pathology for which these novel interventions may be offered.

Although there has been some success in the validation of plasma-based AD biomarkers, it is worth noting that these studies were conducted in carefully selected research cohorts [32]. The participants in this study are regular patients in a memory center with a clinic-based design. Studies have been conducted to address the practical use of AD biomarkers with regard to physician selection of appropriate biomarkers, effective communication to patients, and decision-making of patients and caregivers [33]. Future research can be done in the community setting as well as collaborative studies with other institutions in order to reflect more accurate numbers in regions where amyloid imaging markers are unavailable or unaffordable. The small number (<14) of participants in the NCI/SCI group due to reluctance to testing with very minimal or no symptoms is a limitation that needs to be addressed in future studies. The same limitation could be said of the mixed AD-VaD group where interestingly no difference was found between this and AD and the NCI/SCI groups.

## 5. Conclusion

With the advent of emerging therapies that are targeted at the amyloid pathology, documentation of the patient's amyloid status to confirm etiology of clinical diagnosis is warranted prior to instituting amyloid-based therapy. Based on the current findings, MDS-OA*β* is a simple, noninvasive test and could discriminate between AD and other types of neurodegenerative disorders.

## Figures and Tables

**Table 1:** Clinical characteristics of participants.

	Etiologic diagnosis					*p* value
	NCI/SCI (*N* = 14)	AD (*N* = 72)	Non-AD (*N* = 32)	Mixed AD-VaD (*N* = 29)	Total (*N* = 147)	
Age	66.07 ± 12.26	77.48 ± 7.88	71.47 ± 12.12	78.24 ± 6.94	75.08 ± 10.04	< 0.001
Age groups						
≤59 years	6 (42.9)	1 (1.4)	6 (18.8)	—	13 (8.9)	< 0.001
60-69 years	2 (14.3)	10 (14.1)	7 (21.9)	3 (10.3)	22 (15.1)	
70-79 years	4 (28.6)	28 (39.4)	12 (37.5)	11 (37.9)	55 (37.7)	
≥80 years	2 (14.3)	32 (45.1)	7 (21.9)	15 (51.7)	56 (38.4)	
Sex						
Male	7 (50.0)	24 (33.3)	13 (40.6)	11 (37.9)	55 (37.4)	0.66
Female	7 (50.0)	48 (66.7)	19 (59.4)	18 (62.1)	92 (62.6)	
CDR stages						
Normal	9 (64.3)	—	—	—	9 (6.1)	< 0.001
Very mild	5 (35.7)	45 (62.5)	20 (62.5)	14 (48.3)	84 (57.1)	
Mild	—	17 (23.6)	7 (21.9)	12 (41.4)	36 (24.5)	
Moderate	—	6 (8.3)	4 (12.5)	2 (6.9)	12 (8.2)	
Severe	—	4 (5.6)	1 (3.1)	1 (3.4)	6 (4.1)	
Total	14 (100.0)	72 (100.0)	32 (100.0)	29 (100.0)	147 (100.0)	
PT/C ratio	0.76 ± 0.32	1.22 ± 0.37	0.76 ± 0.37	1.09 ± 0.23	1.05 ± 0.40	< 0.001
PT/C ratio by age groups						
≤59 years	0.76 ± 0.31	1.19 ± 0.00	0.65 ± 0.37	—	0.74 ± 0.34	0.07
60-69 years	0.75 ± 0.21	1.26 ± 0.57	0.89 ± 0.38	1.10 ± 0.14	1.07 ± 0.47	
70-79 years	1.00 ± 0.20	1.16 ± 0.23	0.73 ± 0.40	1.19 ± 0.22	1.06 ± 0.32	
≥80 years	0.29 ± 0.18	1.26 ± 0.41	0.79 ± 0.37	1.01 ± 0.24	1.10 ± 0.42	

Data were presented as mean ± standard deviation or frequency (percentages). NCI: no cognitive impairment; SCI: subjective cognitive impairment; AD: Alzheimer's disease; Non-AD: non-Alzheimer's disease; Mixed AD-VaD: mixed etiology Alzheimer's disease with either vascular cognitive impairment, subcortical ischemic vascular dementia, or cerebrovascular disease.

## Data Availability

The technical appendix, statistical code, and dataset used to support the findings of this study will be made available from the corresponding author upon request.
